# A novel approach to patient safety education: integrating the human factors analysis and classification system (HFACS) to build a culture of safety in medical training

**DOI:** 10.1186/s12909-025-07830-3

**Published:** 2025-10-02

**Authors:** Jiun-Yih Lee, Chien-Hsien Huang, Hsiao-Wei Wang, Shih-Wen Hung, Jui-Ting Chang

**Affiliations:** 1 Center for Quality Management, Shin Kong Wu Ho-Su Memorial Hospital, No. 95, Wenchang Rd., Shilin Dist, Taipei City, 111 Taiwan; 2 Division of Infectious Disease, Department of Internal Medicine, Shin Kong Wu Ho Su Memorial Hospital, Taipei, Taiwan; 3 College of Medicine, Fu-Jen Catholic University, Taipei, Taiwan; 4 Emergency Department, Shin-Kong Wu Ho-Su Memorial Hospital, Taipei, Taiwan; 5 Division of Nephrology, Department of Internal Medicine, Shin Kong Wu Ho-Su Memorial Hospital, Taipei, Taiwan

**Keywords:** HFACS, Medical education, Human factor

## Abstract

**Background:**

This study evaluated the effectiveness of an educational intervention based on the Human Factors Analysis and Classification System (HFACS) in enhancing medical students’ patient safety competencies. The intervention specifically targeted students’ ability to identify human factors and recognize adverse events.

**Methods:**

A quasi-experimental pre–post study was conducted at Shin Kong Wu Ho-Su Memorial Hospital in Taiwan. Forty medical students from two medical schools were recruited; 38 completed both pre- and post-tests for adverse event recognition, and 30 completed both assessments for human factors identification. The 90-minute HFACS-based workshop included theoretical instruction, video-based simulations, and case discussions. Validated questionnaires and structured video analyses were used to evaluate students’ competencies before and after the intervention. Paired *t*-tests were conducted to assess changes.

**Results:**

The intervention significantly improved students’ patient safety competencies. Recognition of reportable adverse events increased from 73.4% (SD = 17.0) to 94.0% (SD = 2.9), *p* < 0.001, Cohen’s *d* = 1.3, 95% CI: 0.15–0.26. Awareness of the importance of reporting rose from 63.9% (SD = 17.2) to 80.4% (SD = 7.8), *p* < 0.001, *d* = 0.9, 95% CI: 0.10–0.23. The total number of identified human factors increased from 5.5 (SD = 2.4) to 36.7 (SD = 14.1), *p* < 0.001, *d* = 2.1, with significant improvements observed across all four HFACS levels.

**Conclusions:**

The HFACS-based educational intervention significantly enhanced medical students’ competencies in recognizing adverse events, understanding the importance of reporting, and identifying human factors across multiple system levels. These findings support the integration of HFACS into medical curricula to promote systemic thinking and foster a proactive safety culture.

## Background

Patient safety has become a cornerstone of contemporary medical education and clinical practice. Since the landmark publication of *To Err is Human* [1], which revealed the systemic origins of medical errors, prevention strategies have taken center stage in healthcare quality improvement. Recent evidence underscores the magnitude of the problem: medical errors are now considered the third leading cause of death in the United States, contributing to over 200,000 preventable deaths annually and harming up to 400,000 hospitalized patients each year [2]. These alarming statistics have eroded public trust in healthcare and highlighted the urgent need for systemic safety reforms. In response, patient safety education is now widely recognized as a critical strategy for improving care quality, with global efforts focused on its integration into medical curricula [3].

Studies have shown that structured safety education improves medical students’ understanding of error causation, disclosure practices, and safety-related attitudes [4]. Simulation-based training and interprofessional education (IPE), in particular, enhance communication and teamwork, thereby reducing clinical errors in high-stakes environments [5–7]. A number of conceptual frameworks support safety education, including the Swiss Cheese Model, which illustrates how latent system failures and active errors align to cause adverse events [8]. The SEIPS model (Systems Engineering Initiative for Patient Safety) adopts a human factors engineering perspective to examine how people, technologies, tasks, and environments interact to influence healthcare outcomes. While models such as Root Cause Analysis (RCA) and Failure Mode and Effects Analysis (FMEA) help analyze incidents and proactively assess risks, they often lack standardized methods for categorizing contributing factors.

In contrast, the Human Factors Analysis and Classification System (HFACS) provides a hierarchical taxonomy that traces the origins of errors across four levels—from individual unsafe acts to latent organizational influences [9, 10]. HFACS complements existing models by offering a structured, systems-based approach that supports both retrospective analysis and proactive educational use. Collectively, these frameworks provide foundational tools for modern patient safety education by facilitating systems thinking and cultural change.

Despite these developments, current curricula often emphasize theoretical knowledge while neglecting the cultivation of students’ ability to recognize human factors—individual and systemic behaviors contributing to unsafe practices [9]. This oversight is concerning, as accurate identification of human factors is essential for both error prevention and safety culture. Additionally, cultural and institutional barriers—such as fear of blame, rigid hierarchies, and lack of support—continue to inhibit reporting behaviors among students and healthcare professionals, even when they recognize adverse events. As prior studies have shown, awareness alone is insufficient; a supportive environment and systems-level understanding are also required to promote meaningful reporting and learning [11]. Medical students frequently struggle to identify latent system vulnerabilities, such as organizational culture or resource allocation, which underlie many preventable events [8]. Moreover, existing programs rarely provide opportunities for students to analyze and report adverse events—despite the critical role this skill plays in risk mitigation and learning [12].

To address these gaps, this study introduces the Human Factors Analysis and Classification System (HFACS) as an educational tool. Originally developed for use in high-risk industries such as aviation, HFACS employs a four-level framework (Unsafe Acts, Preconditions for Unsafe Acts, Supervisory Factors, and Organizational Influences) to systematically trace the root causes of errors [10]. Unlike traditional RCA, HFACS facilitates multi-level analysis that uncovers systemic vulnerabilities—such as resource failures or communication breakdowns—making it especially suited for the complexity of healthcare settings [9]. Although HFACS is increasingly applied in clinical practice, its potential in medical education remains underexplored, particularly in its capacity to develop students’ systemic thinking and ability to generate actionable solutions [4].

This study aims to evaluate the effectiveness of an HFACS-based educational intervention in improving medical students’ competencies related to adverse event reporting and human factor identification. Training rooted in HFACS principles not only strengthens error analysis skills but also fosters a culture of proactive reporting. Specifically, we examined: (1) whether the intervention improves students’ recognition of reportable adverse events; and (2) whether it enhances their ability to identify multi-level human factors. The results aim to inform the design of evidence-based patient safety curricula and support the development of a more resilient safety culture in healthcare.

- Hypothesis 1: An HFACS-based educational intervention will significantly improve students’ recognition of reportable adverse events.
- Hypothesis 2: The intervention will significantly enhance students’ ability to identify human factors contributing to medical errors across all four HFACS levels.

## Methods

### Study design and sample

This study adopted a quasi-experimental pre–post design to evaluate the effectiveness of a Human Factors Analysis and Classification System (HFACS)-based educational intervention in enhancing medical students’ patient safety competencies. The intervention was implemented at Shin Kong Wu Ho-Su Memorial Hospital (SKH), an 829-bed medical center located in northern Taiwan. The study comprised three phases:

- Pre-test Phase: Participants completed a questionnaire assessing their knowledge of reportable adverse events and viewed a video simulating a clinical scenario. They were instructed to identify potential safety issues to evaluate baseline competence in recognizing human factors.
- Workshop Phase: A structured workshop was conducted, focusing on adverse event reporting and the application of the HFACS framework. Instruction centered on the systematic identification of unsafe human factors within healthcare processes.
- Post-test Phase: Participants repeated the pre-test questionnaire and video exercise to assess improvement in human factors identification.

Following the post-test, students participated in a structured debriefing session led by HFACS-certified instructors. They re-examined the video case using the HFACS framework, discussing contributing factors at all four levels. Although conducted after formal data collection, the session aimed to reinforce learning, enhance systems thinking, and consolidate error analysis skills.

Eligible participants were fifth- and sixth-year medical students from Fu Jen Catholic University (northern Taiwan) and China Medical University (central Taiwan). Fifth- and sixth-year medical students were specifically selected because they had already completed foundational clinical rotations and possessed sufficient clinical exposure to comprehend adverse event scenarios and human factors concepts, ensuring meaningful engagement with the workshop content. A total of 40 students were recruited via convenience sampling, representing the entire eligible cohort. Inclusion criteria required completion of both pre- and post-tests. Consequently, 38 students were included in the analysis of adverse event awareness, and 30 in the analysis of human factors identification.

### Workshop design and study instruments

#### Workshop design

The 90-minute workshop was collaboratively developed by the first and corresponding author—a certified HFACS instructor at the Center for Quality Management—and a senior attending physician in General Medicine responsible for undergraduate medical education. The design process ensured alignment with both HFACS learning objectives and clinical relevance. This study has been approved and funded by the research project of Shin Kong Wu Ho-Su Memorial Hospital (2025SKHAND016). The workshop comprised three instructional modules:

I. Fundamental Concepts of Patient Safety: An overview of patient safety principles was provided, emphasizing the importance of minimizing harm and promoting quality care.
II. Recognition of Reportable Adverse Events: Thirteen categories of adverse events, as defined by Taiwan’s Joint Commission on Hospital Accreditation Patient Safety Reporting System (TPR), were introduced. Real-world case examples were used to contextualize learning.
III. Introduction to HFACS and Its Proactive Application in Identifying Human Factors: The four hierarchical levels of HFACS—Unsafe Acts, Preconditions for Unsafe Acts, Supervisory Factors, and Organizational Influences—were explained in detail. A structured attribution guide was used to support systematic analysis. Illustrative cases from previous research [13] were incorporated to demonstrate practical applications.

### Study instruments

#### Recognition of adverse events

A structured questionnaire, adapted from previous research [11], was used to assess students’ ability to recognize adverse events. It consisted of two sections: (1) *Recognition of Reportable Adverse Events*: Seventeen items evaluated students’ knowledge of events warranting reporting (e.g., “Which of the following should be reported as an adverse event?”). (2) *Importance of Reporting*: Five items assessed the perceived value of reporting (e.g., “Proactively reporting adverse events facilitates learning and prevents recurrence.”).

Scores were calculated as the percentage of correct responses. Content validity was established via expert panel review, comprising three senior physicians and two patient safety specialists. Pilot testing with a separate cohort yielded strong internal consistency (Cronbach’s α = 0.92).

#### Human factors identification

To evaluate students’ ability to recognize human factors, a standardized video adapted from real-life cases was utilized. This video, part of the patient safety education training program at SKH, depicted a fatal medication administration error (route error) and highlighted common human factors contributing to the event. The video storyline incorporated various elements of the HFACS framework, including: Procedural violations, communication and coordination failures, improper task execution, physiological and psychological conditions, and organizational resource management.

These elements comprehensively covered all four levels of the HFACS framework. An open-ended questionnaire was used to assess students’ ability to identify human factors depicted in the video. A sample question included: “Please list the issues you identified in the video.” Two HFACS-certified analysts, accredited by the Taiwan Joint Commission on Hospital Accreditation, reviewed and categorized the responses according to the HFACS framework. The number of human factors identified by each student was recorded as a continuous variable for further analysis.

The video case used to assess human factors recognition was adapted from a real sentinel event and validated through iterative review by certified HFACS experts. Two independent raters, both certified in HFACS by the Taiwan Joint Commission on Hospital Accreditation, coded the open-ended responses. Inter-rater reliability was high (Cohen’s kappa = 0.88), indicating strong agreement in categorizing student-identified human factors across the four HFACS levels.

### Statistical methods

Paired-sample *t* tests were conducted to compare pre- and post-test scores for both outcome measures. Assumptions of normality and homogeneity of variance were verified using the Shapiro–Wilk test, respectively (all *p* > 0.05), supporting the use of parametric testing. Two-tailed *p*-values < 0.05 were considered statistically significant. All analyses were performed using SPSS software (version 27.0; IBM Corp., Armonk, NY, USA).

## Results

### Sample characteristics

A total of 40 medical students participated in the study, including 19 fifth-year (47.5%) and 21 sixth-year (52.5%) students. Among them, 38 students completed both the pre- and post-test for the adverse event awareness assessment (95.0% completion rate), while 30 students completed both the pre- and post-tests for the human factors identification task (75.0% completion rate).

### Recognition of adverse events

To evaluate Hypothesis 1, changes in students’ ability to recognize reportable adverse events and their awareness of the importance of reporting were examined following the HFACS-based educational intervention.

**Fig. 1:**
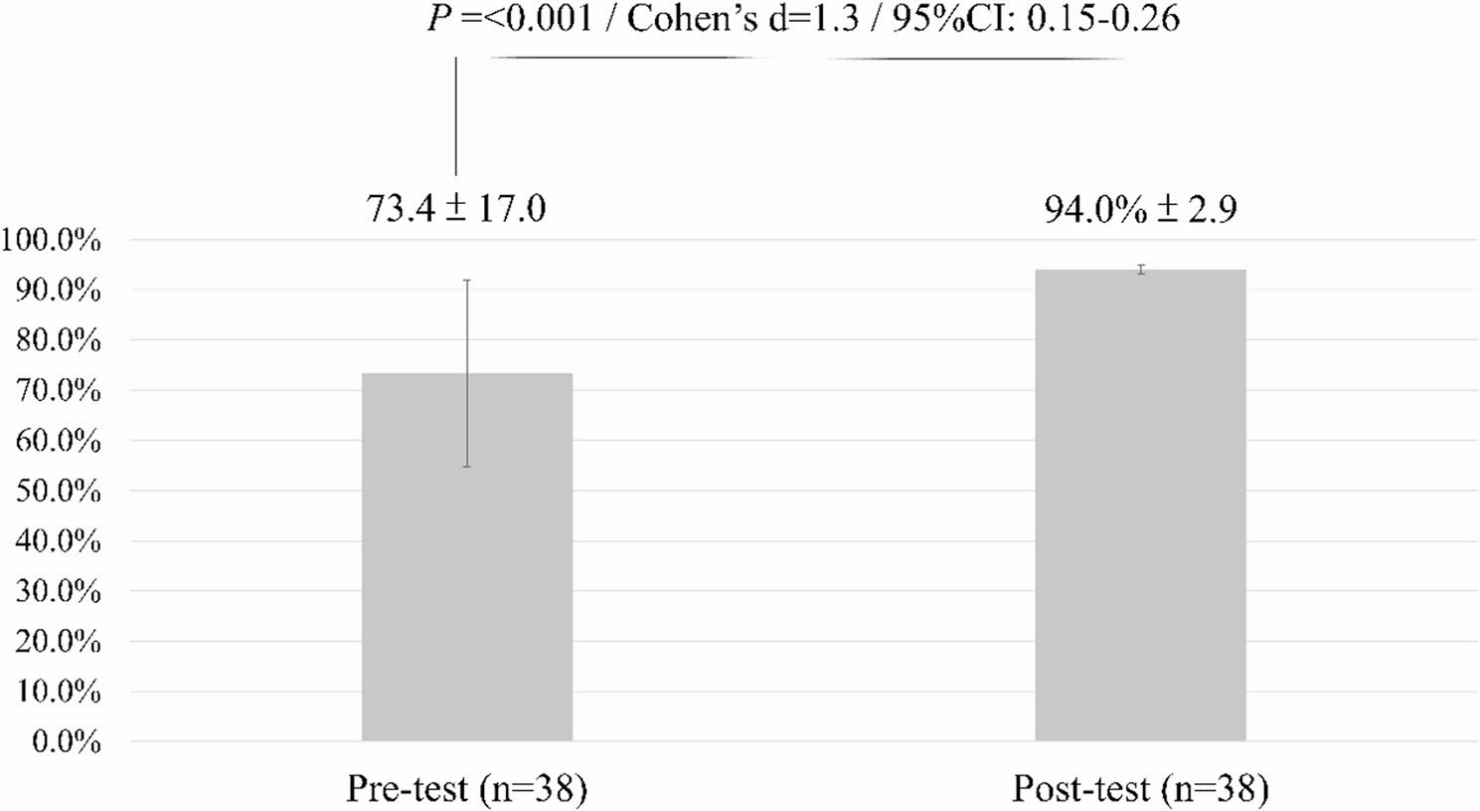
Recognition of reportable adverse events

As illustrated in Fig. 1, the mean recognition score for reportable adverse events increased significantly from 73.4% (SD = 17.0) at pre-test to 94.0% (SD = 2.9) at post-test (*p* < 0.001), corresponding to a large effect size (Cohen’s *d* = 1.3; 95% CI: 0.15–0.26).

**Fig. 2:**
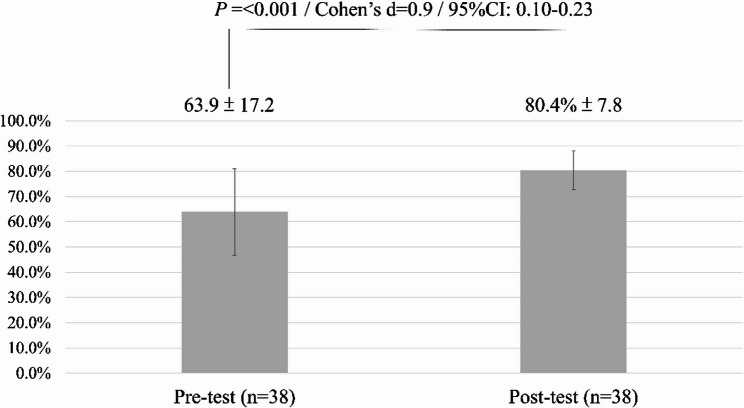
Recognition of the Importance of Reporting

Likewise, students’ awareness of the importance of reporting improved significantly, as shown in Fig. 2. The mean score rose from 63.9% (SD = 17.2) to 80.4% (SD = 7.8), *p* < 0.001, with a large effect size (Cohen’s *d* = 0.9; 95% CI: 0.10–0.23).

These findings support Hypothesis 1, indicating that the HFACS-based intervention significantly enhanced students’ recognition of reportable adverse events and increased their appreciation of reporting’s role in promoting clinical safety.

### Ability to identify human factors

To evaluate Hypothesis 2, students’ ability to identify human factors contributing to medical errors was compared before and after the intervention. The total number of identified human factors increased markedly from a mean of 5.5 (SD = 2.4) at pre-test to 36.7 (SD = 14.1) at post-test, *p* < 0.001, with a very large effect size (Cohen’s *d* = 2.1; 95% CI: 25.6–36.6).

Significant gains were observed across all four HFACS levels—Unsafe Acts, Preconditions for Unsafe Acts, Supervisory Factors, and Organizational Influences—with effect sizes ranging from *d* = 1.1 to 2.4. The most pronounced improvements were noted at Level 2 (Preconditions) and Level 1 (Unsafe Acts). Detailed results by HFACS level and subcategory, including effect sizes and confidence intervals, are presented in Table 1.

**Table 1:** Recognition of human factors

HFACS Categories	Pre-test Mean (SD) (*n* = 30)	Post-test Mean (SD) (*n* = 30)	Cohen’s d	*p*	95% Confidence Interval	
					Lower Bound	Upper Bound
Total	5.5 (2.4)	36.7 (14.1)	2.1	< 0.001	25.6	36.6
Level 1: Unsafe Acts	2.6 (0.8)	11.1 (3.4)	2.4	< 0.001	7.2	9.9
Decision Errors	0.4 (0.7)	2.8 (0.9)	2.4	< 0.001	2.0	2.8
Skill-based Errors	1.1 (0.7)	2.7 (0.9)	1.4	< 0.001	1.2	2.1
Perceptual Errors	0.1 (0.3)	1.6 (1.1)	1.3	< 0.001	1.1	1.9
Routine Violations	0.2 (0.6)	2.1 (0.8)	2.0	< 0.001	1.5	2.3
Exceptional Violations	0.7 (0.6)	1.8 (1.0)	1.0	< 0.001	0.7	1.5
Level 2: Preconditions for Unsafe Acts	2.1(1.1)	15.3 (5.5)	2.2	< 0.001	11.0	15.4
Physical Environment	0.4 (0.5)	2.3 (1.1)	1.7	< 0.001	1.5	2.4
Tools and Technology	0.0 (0.2)	1.0 (1.0)	1.0	< 0.001	0.6	1.4
Task	0.1 (0.4)	2.1 (1.1)	1.6	< 0.001	1.4	2.4
Psychological State	0.4 (0.5)	2.5 (1.1)	1.8	< 0.001	1.7	2.5
Physiological State	0.0 (0.0)	1.2 (1.4)	0.9	< 0.001	0.7	1.7
Fitness	0.0 (0.0)	0.7 (1.0)	0.7	0.001	0.4	1.1
Communication	0.7 (0.7)	1.9 (0.4)	1.5	< 0.001	0.9	1.5
Coordination	0.5 (0.5)	1.7 (0.8)	1.5	< 0.001	0.9	1.6
Leadership	0.0 (0.0)	1.8 (1.0)	1.8	< 0.001	1.4	2.1
Level 3: Supervisory Factors	0.6 (0.6)	6.0 (2.9)	2.0	< 0.001	4.4	6.5
Inadequate Supervision	0.6 (0.5)	1.8 (1.3)	1.0	< 0.001	0.8	1.7
Planned Inappropriate Operations	0.0 (0.2)	1.5 (0.8)	1.9	< 0.001	1.2	1.8
Failure to Correct Known Problems	0.0 (0.0)	1.3 (0.9)	1.4	< 0.001	0.9	1.6
Supervisory Violations	0.0 (0.0)	1.4 (0.7)	2.1	< 0.001	1.2	1.7
Level 4: Organizational influences	0.3 (0.8)	4.2 (3.4)	1.1	< 0.001	2.6	5.3
Organizational Culture	0.1 (0.3)	1.4 (1.3)	1.0	< 0.001	0.9	1.8
Process Management	0.0 (0.0)	1.8 (1.3)	1.4	< 0.001	1.3	2.3
Resource Management	0.0 (0.2)	1.0 (1.2)	0.8	< 0.001	0.5	1.5

Collectively, these results confirm that the HFACS-based workshop effectively enhanced students’ capacity to identify multi-level human factors underlying medical errors.

## Discussion

### Statement of principal findings

This study demonstrates that an educational intervention based on the Human Factors Analysis and Classification System (HFACS) significantly enhanced medical students’ patient safety competencies. Specifically, the results support Hypothesis 1, as students showed marked improvement in recognizing reportable adverse events. Hypothesis 2 was also supported, with significant gains observed in the identification of human factors across all four HFACS levels—Unsafe Acts, Preconditions for Unsafe Acts, Supervisory Factors, and Organizational Influences.

Furthermore, the intervention heightened students’ awareness of the importance of adverse event reporting, establishing a solid foundation for fostering a culture of patient safety. These results suggest that HFACS-based training not only equips students with the analytical skills to systematically identify errors but also encourages proactive engagement with error reporting systems in clinical practice. This engagement fosters transparency, accountability, and continuous learning, strengthening a sustainable safety culture in healthcare.

### Interpretation within the context of the wider literature

These results align with prior studies emphasizing the value of patient safety education in medical training. Previous research has highlighted the effectiveness of simulation-based and interactive approaches in enhancing knowledge, skills, and safety attitudes among medical students [5, 7]. Notably, this study represents the first application of the HFACS framework in undergraduate medical education, addressing a critical gap identified by Nie et al. [4], who underscored the lack of focus on human factors analysis in traditional curricula.

The observed improvements across all HFACS levels affirm the framework’s educational utility in promoting systemic error analysis. Students were able to identify contributing factors not only at the individual level but also at supervisory and organizational levels—underscoring HFACS’s ability to cultivate multi-layered thinking. These findings extend existing evidence of HFACS’s value in retrospective healthcare error analysis [9, 10] by demonstrating its potential in proactive training environments.

However, despite overall improvement, students identified fewer organizational-level factors (mean = 4.0) compared to Level 1 Unsafe Acts (mean = 10.6), suggesting limited capacity to recognize latent, high-order system issues such as resource allocation or process failures. This echoes critiques of Root Cause Analysis (RCA), which often fails to address deeper organizational flaws [8]. Addressing this challenge requires deliberate curricular strategies that emphasize higher-level system dynamics, in line with Leape’s assertion that addressing systemic safety issues necessitates comprehensive education and cultural change [14].

Although simulation-based training improves performance and safety knowledge [5, 7], it is increasingly recognized that without structured debriefing and opportunities for reflective learning, this training may not sustain behavioral change. In this study, the HFACS framework offered students a structured lens through which to interpret simulated scenarios, deepening reflection and enhancing knowledge transfer to real-world settings. While the debriefing session occurred after data collection, it served as a valuable consolidation activity to reinforce systemic thinking.

### Implications for sustainable education design

These findings underscore the importance of designing sustainable safety education models. While short-term gains in safety attitudes are common, integrating HFACS into longitudinal curricula may foster enduring improvements in clinical reasoning and reporting behavior [5]. Moreover, embedding HFACS into interprofessional simulation training could facilitate collaborative error analysis, promoting shared understanding and team-based solutions.

For broader scalability and practical integration, routine clinical debriefings grounded in real patient cases offer a compelling alternative to resource-intensive simulation centers. These case-based reflections can be seamlessly embedded into clinical workflows—such as shift handovers, safety huddles, or morbidity and mortality reviews—with minimal operational disruption. A recent study by Paquay et al. [15] demonstrated that debriefings provide insights into systemic vulnerabilities comparable to those derived from formal incident reporting and can effectively enhance teamwork and safety culture in real time.

In this context, HFACS-guided debriefings present a practical avenue to integrate systems thinking into everyday clinical practice. By anchoring learning in real-world contexts, this approach bridges the gap between education and practice, fostering proactive problem-solving and continuous improvement.

Although HFACS remains in the early stages of application in medical education, this study provides empirical evidence supporting its feasibility and value in undergraduate training. Its structured, holistic approach equips students with critical thinking and systemic awareness—capabilities often lacking in traditional curricula. Future programs should consider combining RCA and HFACS frameworks to offer a more comprehensive understanding of error causation, particularly at the organizational level.

Embedding HFACS into core curricula and simulation exercises can bridge the disconnect between theoretical instruction and clinical realities. Active student participation in safety improvement initiatives may further enhance experiential learning and cultivate a proactive safety mindset.

### Strengths and limitations

This study offers several strengths, including the use of validated instruments, structured video assessments, and a clear pre–post design. However, limitations remain. The absence of a control group introduces potential bias, as improvements may be attributable to general educational effects rather than the specific impact of the HFACS framework. Partial data from some participants may also affect internal validity. Moreover, the modest sample size, drawn from only two medical institutions in Taiwan, may limit generalizability. The short follow-up duration precludes assessment of long-term retention or behavioral change. Finally, the lack of interprofessional education (IPE) components limits the ability to evaluate team-based learning outcomes. Future studies should adopt controlled, longitudinal designs and explore the integration of HFACS into IPE and RCA training to strengthen system-based learning and reporting behaviors across healthcare teams.

## Conclusion

This study demonstrates that an educational intervention based on the Human Factors Analysis and Classification System (HFACS) significantly improved medical students’ patient safety competencies. Students showed enhanced ability to recognize reportable adverse events and a deeper appreciation of the importance of reporting. Moreover, they gained substantial skills in identifying human factors across all four HFACS levels, particularly at the levels of Unsafe Acts and Preconditions for Unsafe Acts.

These findings support integrating HFACS into undergraduate medical education as a structured method to cultivate systems thinking and promote proactive safety behaviors. The relatively lower identification of organizational-level factors highlights the need for longitudinal, experiential strategies to build students’ capacity to detect systemic risks. Future programs should embed HFACS into both classroom and clinical settings—including structured debriefings and interprofessional simulation training—to support sustained learning and foster a culture of safety in practice.

## Data Availability

The data underlying this article cannot be shared publicly due to privacy issues. However, they may be provided upon reasonable request. The data can be obtained from the first author, Jiun-Yih Lee (E-mail: m511098001@tmu.edu.tw).

## Abbreviations

HFACS: Human Factors Analysis and Classification System
RCA: Root Cause Analysis
TPR: Taiwan’s Joint Commission on Hospital Accreditation Patient Safety Reporting System
IPE: Inter Professional Education
